# New PCR primers for metabarcoding environmental DNA from freshwater eels, genus *Anguilla*

**DOI:** 10.1038/s41598-019-44402-0

**Published:** 2019-05-28

**Authors:** Aya Takeuchi, Tetsuya Sado, Ryo O. Gotoh, Shun Watanabe, Katsumi Tsukamoto, Masaki Miya

**Affiliations:** 1 Graduate School of Bioresource Sciences, Nihon University, 1866 Kameino, Fujisawa, Kanagawa 252-0880 Japan; 2 Department of Ecology and Environmental Sciences, Natural History Museum and Institute, 955-2 Aoba-cho, Chuo Chiba, 260-8682 Japan; 3 Department of Fisheries, Faculty of Agriculture, Kindai University, 3327-204 Nakamachi, Nara, 631-8505 Japan; 4 Present Address: Department of Aquatic Bioscience, Graduate School of Agricultural and Life Sciences, The University of Tokyo, 1-1-1 Yayoi, Bunkyo, Tokyo, 113-8657 Japan

**Keywords:** Genetic markers, Molecular biology, Molecular ecology, Marine biology

## Abstract

Freshwater eels of the genus *Anguilla* comprise 16 species that include three subspecies and are characterized by their unique catadromous life cycles. Their life histories and nocturnal life styles make it difficult to observe them in freshwater and marine habitats. To investigate their distribution and ecology in aquatic environments, we developed new PCR primers for metabarcoding environmental DNA (eDNA) from *Anguilla*. The new primers (MiEel) were designed for two conserved regions of the mitochondrial ATP6 gene, which amplify a variable region with sufficient interspecific variations ranging from five to 22 nucleotide differences (one to three nucleotide differences between three subspecies pairs). We confirmed the versatility of the MiEel primers for all freshwater eels using tissue DNA extracts when analyzed separately. The metabarcoding combined with the MiEel primers using mock communities enabled simultaneous detection of *Anguilla* at the species level. Analysis of eDNA samples from aquarium tanks, a controlled pond and natural rivers demonstrated that the MiEel metabarcoding could successfully detect the correct *Anguilla* species from water samples. These results suggested that eDNA metabarcoding with MiEel primers would be useful for non-invasively monitoring the presence of the endangered anguillid eels in aquatic environments where sampling surveys are difficult.

## Introduction

Environmental DNA (eDNA) can be extracted from environmental samples such as soil, water or air without isolating any target species^1^. Aquatic organisms release various quantities of eDNA through metabolic waste, damaged tissue, or sloughed skin cells in their environments^2^. Ficetola *et al*.^3^ was the first study to present an eDNA analysis that could detect the American bull frog *Lithobates catesbeianus* as invasive species in controlled environments and natural wetlands. Subsequently, the eDNA analysis has been applied to the detection of a single or a few rare, endangered and closely related species in aquatic ecosystems^3–7^. Furthermore, eDNA analysis using high-throughput next-generation sequencing (NGS) has enabled simultaneous multiple-species identification from a single environmental sample, which has been called “eDNA metabarcoding”, in aquaria, mesocosms, natural rivers and coastal areas^8–10^. The efficiency of eDNA metabarcoding was comparatively better than or at least as good as conventional survey methods such as net sampling, snorkeling observation, and bottom trawling^8,10,11^. eDNA metabarcoding is non-invasive and is expected to be a sensitive, cost- and time-effective method to estimate the distribution and biodiversity of various aquatic organisms.

**Figure 1:**
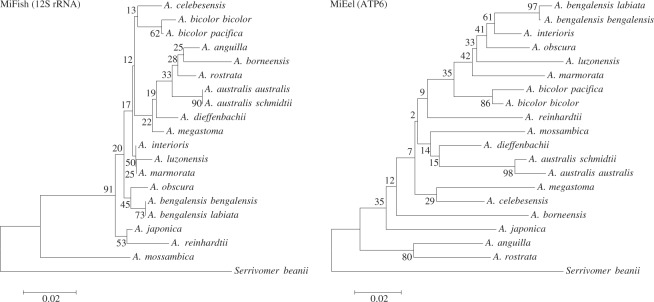
Neighbor-joining tree of 19 species and subspecies the genus *Anguilla* plus *Serrivomer beanii* as an out-group based on the amplified regions of MiFish^9^ (12S rRNA) and MiEel (ATP6 gene). Distance was calculated using the Kimura two parameter model with all sequences containing gaps being eliminated, and numerals beside the internal branches are bootstrap probabilities of 300 replicates. This analysis of phylogenetic tree was conducted in MEGA6^35^.

eDNA metabarcoding requires a genetic marker with suitable taxonomic resolution and an appropriate methodology using NGS. For example, Miya *et al*.^9^ developed universal PCR primers, named MiFish, with high fish taxonomic resolution and detected more than 230 subtropical marine fish species from aquarium tanks and coral reefs. Valentini *et al*.^10^ identified a maximum of 19 fish species from water samples using another set of universal primers developed by an ECOPRIMERS software (see ref.^12^). However, the eDNA metabarcoding studies using those developed universal primers that amplified short fragments within the 12S ribosomal RNA (12S rRNA) could not always distinguish all target species^9,10^. Indeed, eDNA metabarcoding using MiFish primers could not assign amplicon sequences to *Sebastes* spp, *Takifugu* spp^13^ and freshwater eels *Anguilla* (Fig. 1). The low taxonomic resolution in some target species resulted from highly similar sequences among closely related species and a lack of their sequences in the reference databases^9,10,14^.

There is great concern about the conservation status of the catadromous freshwater eels of the genus *Anguilla*, which are one group of fishes that can be difficult to study using conventional sampling methods. Eel populations have dramatically declined in recent decades apparently due to multiple factors such as habitat loss, overexploitation and ocean-atmosphere changes^15^. Most *Anguilla* are now classified at various levels of concern by the IUCN Red List^15,16^. However, their life histories hamper their conservation and management. They are famous for their remarkable migrations between ocean spawning areas and freshwater growth habitats^17^. A survey in their oceanic spawning areas requires a research vessel, and major efforts to use large nets or trawls to collect their eggs, larvae and adults^18,19^. In freshwater, juveniles of *A. anguilla* and *A. japonica* spend most of their time hiding in refuges during daytime^20,21^. Telemetry tracking studies indicated that *A. anguilla*, *A. australis*, *A. dieffenbachii*, and *A. rostrata* were active mostly at night in river systems^22–24^. The remarkable migrations and fossorial life styles make it difficult to observe them in aquatic environments and monitor their presence or absence. A species-specific method to detect DNA from fish stomachs and eDNA has been already used for *A. anguilla*^25,26^ and *A. japonica*^27^ to begin to overcome these difficulties.

The aquatic environment suspends DNA source materials, enabling it to be sampled and detected even for species that are difficult to observe or collect^4^. Assuming that eDNA metabarcoding can effectively detect multiple species presence without visual observation, it will complement a monitoring in marine and freshwater habitats of all freshwater eels whose populations have rapidly decreased. The objective of this study was to develop new PCR primers at the species or subspecies level for eDNA metabarcoding of the genus *Anguilla*. Additionally, the performance of newly developed primers (herein referred to as MiEel) was tested using tissue-derived DNA extracts from 19 freshwater eels and eDNA samples from aquarium tanks, a controlled pond and natural rivers.

## Methods

### Primer design

For analysis of metabarcoding eDNA, new primers were designed following three requirements: (1) a target amplicon with less than 200 bp is desirable because the eDNA will often be degraded, (2) the amplified regions include sufficient interspecific differences for all target species and (3) conserved regions (20–30 bp) across all target species are located at both ends of the short variable regions to simultaneously amplify targeted sequences^9,10,12^. The search for a region that satisfied the above requirements was performed on mitochondrial DNA (mtDNA) sequences with a high copy number per cell. The complete mtDNA for 19 species and subspecies of the genus *Anguilla* (DDBJ/EMBL/GenBank accession number AB038556, AP007233–AP007249, AB469437 from Minegishi *et al*.^28,29^) was used for the primer design. All the sequences were aligned using MAFFT v. 6.956^30^, and the aligned sequences were visually inspected with MESQUITE v. 2.75^31^ to locate the short variable region flanked by two conserved regions. Melting temperatures (T_m_) of the designed primers were calculated using a nearest neighbor method implemented in OligoCalc, and G/C contents were also evaluated^32^.

A WebLogo^33^ was used to explore the similarity between the sequences of the newly designed primers and species of the anguilliforms including *Anguilla*. We downloaded whole mitogenome sequences of 19 other closely related anguilliforms that determined by Inoue *et al*.^34^ that conducted a phylogenetic analysis. The 19 sequences were registered specimens with voucher numbers in the DDBJ. To avoid wrong taxonomic assignment of sequences, we also calculated pairwise distances between *Anguilla* and the anguilliforms for the sequences that would be amplified by the MiEel primers using MEGA6^35^.

### Test of primers with extracted DNA

To examine whether the designed primers amplify the target regions for all freshwater eels, the performance was tested using extracted DNA from a single individual of each 19 species and subspecies of *Anguilla*. Total DNA was extracted from each tissue (muscle, liver or fin), that was preserved in 99% ethanol for more than 10 years, using DNeasy Blood & Tissue Kits (Qiagen, Hilden, Germany) with an elution volume of 100 µl. Species identification of the tissue samples used in this study were carefully morphologically and genetically conducted previously^36^, which assured correct taxonomic assignment of sequences to each species of *Anguilla*. DNA concentrations were measured with a NanoDrop Lite spectrophotometer (Thermo Fisher Scientific, DE, USA), and the extracted DNA was diluted to 5 ng μl^−1^ using sterile distilled H_2_O. PCR was carried out with 30 cycles of a 7.67 µl reaction volume containing 2.29 µl of sterile distilled H_2_O, 3.82 µl of 2 × Gflex PCR buffer (Takara, Otsu, Japan), 0.38 µl of each primer (5 µM), 0.16 µl of Tks Gflex DNA polymerase (Takara, Otsu, Japan) and 0.64 µl of the diluted DNA extracts. The thermal cycle profile after an initial denaturation at 94 °C for 1 minute was as follows: denaturation at 98 °C for 10 s, annealing at 50 °C for 10 s and extension at 68 °C for 10 s with the final extension at the same temperature for 7 minutes. The PCR products were electrophoresed on 2% agarose gel (L03; Takara, Otsu, Japan) to check the amplifications. The PCR products were purified using Exo Sap-IT (USB, OH, USA) to remove redundant dNTPs, primers and nucleotides. Direct sequencing of the purified PCR products was performed with the ABI 3130xl Genetic Analyzer (Life Technologies, CA, USA) and dye-labelled terminators (BigDye terminator v. 1.1; Applied Biosystems, CA, USA). The DNA sequences were edited and assembled by GENETYX-MAC v. 17 (Genetyx, Tokyo, Japan) and registered in the DDBJ/EMBL/NCBI database (accession number LC198042–198060).

### Library preparation and MiSeq sequencing with mock communities

To test whether the metabarcording approach using MiEel primers can distinguish the 19 species freshwater eels, six types of mock community were prepared using tissues-derived DNA extracts (diluted in 1.5 ml tubes to 5 ng μl^−1^) of different combinations of the *Anguilla* species. Six mock communities were subjected to the first-round PCR (1st PCR) and the second-round PCR (2nd PCR) in order to append amplified sequences with three kinds of adaptor sequence: (1) primer-binding sites for sequencing, (2) dual-index sequences to distinguish amplicons and (3) sequences for binding to the flowcells of the Illumina MiSeq (Illumina, CA, USA). The 1st PCR was carried out with 35 cycles of a 12 µl reaction volume containing 6.0 µl of 2 × KAPA HiFi HotStart ReadyMix (KAPA Biosystems, MA, USA), a final concentration of 0.3 µM of each MiEel primer, 2.6 µl of sterile distilled H_2_O and 2.0 µl of each mock community. The thermal cycle profile after an initial denaturation at 95 °C for 3 minutes was as follows: denaturation at 98 °C for 20 s, annealing at 65 °C for 15 s and extension at 72 °C for 15 s with the final extension at the same temperature for 5 minutes. The 1st PCR products were purified using a MinElute Gel Extraction Kit (Qiagen, Hilden, Germany). Subsequently, the purified products were quantified using TapeStation 2200 (Agilent, Tokyo, Japan), diluted to 0.1 ng µl^−1^ using sterile distilled H_2_O, and then used as a template for the 2nd PCR.

The 2nd PCR was conducted with 12 cycles of a 12 µl reaction volume containing 6.0 µl of 2 × KAPA HiFi HotStart ReadyMix, 0.7 µl of each primer (5 µM), 3.6 µl of sterile distilled H_2_O and 1.0 µl of the template. Different combinations of dual-index sequences (chosen from A/D501−508 for forward primers and A/D701−712 for reverse primers from Miya *et al*.^9^) were used for different templates in the 2nd PCR. The thermal cycle profile after an initial denaturation at 95 °C for 3 minutes was as follows: denaturation at 98 °C for 20 s, annealing and extension combined at 72 °C (shuttle PCR) for 15 s with the final extension at the same temperature for 5 minutes. 1st PCR and 2nd PCR blanks were included with each PCR run to monitor contamination.

All the libraries containing the target regions and the three adapter sequences were mixed in equal volume, and the pooled libraries were size-selected from approximately 340 bp using 2% E-Gel Size Select agarose gel (Invitrogen, CA, USA). The concentration of the size-selected libraries was measured using a Qubit dsDNA HS assay Kit and a Qubit Fluorometer (Life Technologies, CA, USA). The libraries were sequenced on the Miseq platform using a MiSeq v2 Reagent Kit for 2 × 150 bp PE (Illumina, CA, USA) following the manufacturer’s protocol.

### Water sampling and filtration

An on-site filtration method developed by Miya *et al*.^37^ was employed to collect fresh eDNA. Disposable gloves were worn and changed between each sample. After collecting surface water using a bucket, the water was drawn into a disposable 50 ml syringe (Terumo, Tokyo, Japan) and then pushed through a 0.45 µm Sterivex filter cartridge (Millipore, MA, USA). This step was repeated until 500 ml water volume was filtered. A RNAlater of 1.6 ml (Thermo Fisher Scientific, DE, USA) was added into the cartridge after the filtration to prevent eDNA degradation. The bucket was sterilized with 0.5% bleach and thoroughly prewashed with tap water or freshwater before reuse.

For aquarium tanks and a pond, water of 500 ml was filtered from each of two aquarium tanks (120 × 45 × 40 cm; water volume = 205 l) that each contained one species (*A. bicolor bicolor*, *A. australis* sp., respectively) on 22 August 2017. The total length of the two eels in each aquarium was approximately 70 cm. The two tanks were not sterilized because they were used as displays at the International Eel Laboratory, Miyazaki Prefecture, Japan. On the same day, 500 ml of surface water was filtered from the corner of the controlled pond (45 × 20 × 1 m, water volume = 850,000 l) containing 20 individuals of *A. japonica* with the total length ranging from 52 to 80 cm.

**Figure 2:**
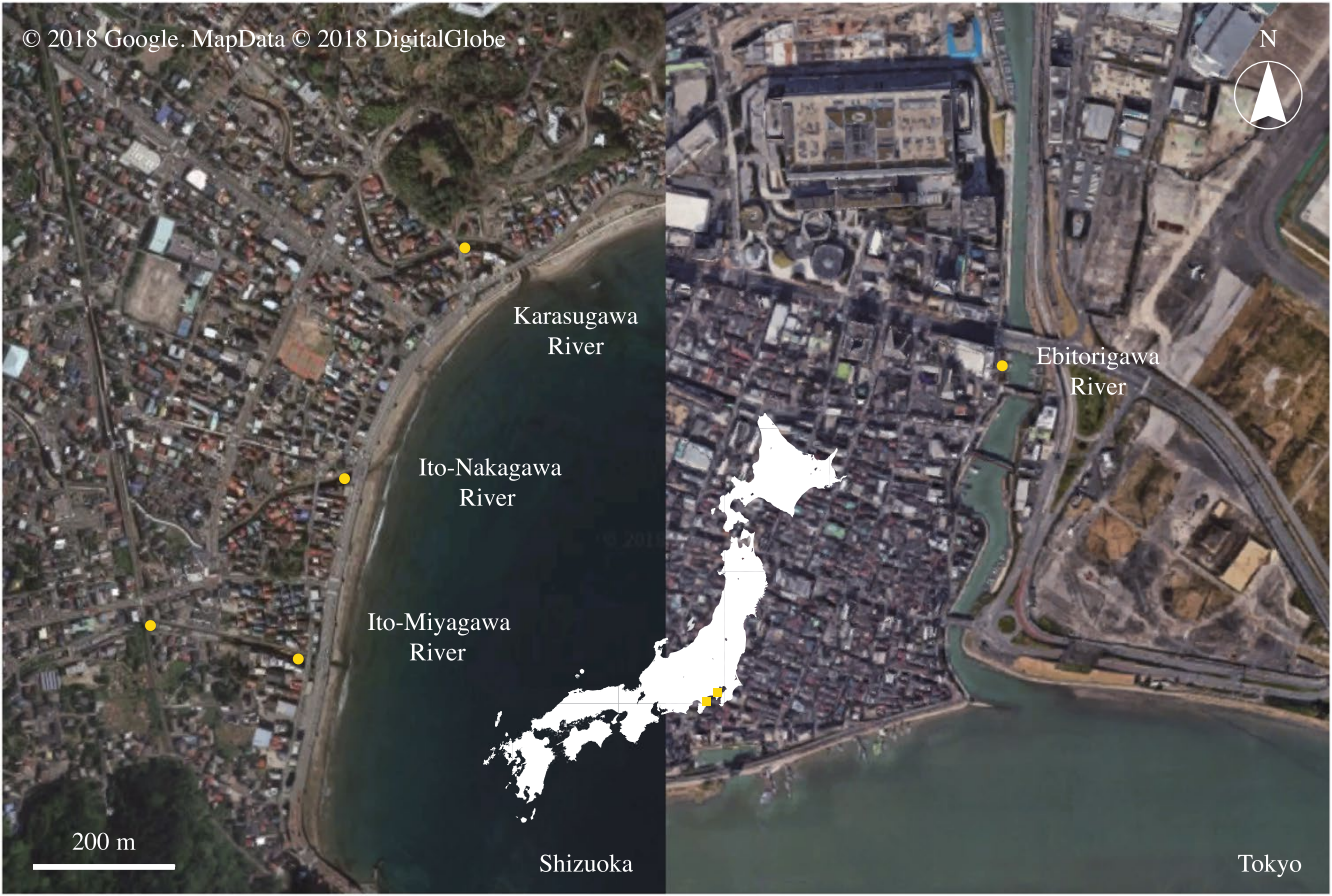
Water sampling sites (yellow circles). The Karasugawa River (35.008°N, 139.085°E), Ito-Nakagawa River (35.005°N, 139.083°E), and Ito-Miyagawa River (35.002°N, 139.082°E; 35.003°N, 139.080°E) in Shizuoka and the Ebitorigawa River (35.550°N, 139.752°E) in Tokyo. The map overlay shows the sampling locations (yellow squares) within Shizuoka and Tokyo Prefectures within central Japan. Panels were made from imagery obtained from the Google Earth program.

For natural rivers, 500 ml of surface water was filtered from the Ebitorigawa River (35.550°N, 139.752°E), the upper estuaries of the Ito-Miyagawa River (35.002°N, 139.082°E; 35.003°N, 139.080°E), Ito-Nakagawa River (35.005°N, 139.083°E) and Karasugawa River (35.008°N, 139.085°E) in Tokyo or Shizuoka, Japan on 10 September 2017 (Fig. 2). All of the rivers are known as suitable habitats for *A. japonica*. Each freshwater sample was carefully filtered without entering the river to avoid possible contamination. A filtration blank was created by filtering 500 ml of sterile distilled H_2_O in the same manner as eDNA samples after the end of the water sampling each day. Ten filter cartridges (two from tanks, one from the controlled pond, five from the rivers and two filtration blanks) were transported to the laboratory in a cooler with ice, and then kept at −20 °C for about 20 days until eDNA extractions.

### Library preparation and MiSeq sequencing with eDNA samples

Strict clean-lab procedures were adopted when eDNA samples were analyzed to minimize contamination. Prior to eDNA library preparations, work-space and equipment were totally sterilized, filter pipet tips were used, and separation of pre- and post-PCR was carried out to safeguard against contamination. Extraction, the 1st PCR, and the 2nd PCR blanks were processed alongside the eDNA samples to monitor contamination.

eDNA was extracted from all cartridges using a DNeasy Blood & Tissue Kit (Qiagen) following the manufacture’s protocol and the method developed by Miya *et al*.^9,37^. All eDNA extracts including blanks were used for multiplex PCR using fish universal primers MiFish (from Miya *et al*.^9^) plus MiEel primers for a correct assignment of *Anguilla*. When the 1st PCR was multiplexed, the final concentration of each primer was 0.3 µM, and sterile distilled H_2_O was added up to the total reaction volume of 12 µl. Eight PCR replications were made per one of each eDNA extract (except for all blanks) for the 1st PCR to avoid missing detection of target species that are actually present^38^. The eight PCR replications of each extract were pooled, and then the pooled product was purified in the elution volume of 12 µl using a MiniElute Gel Extraction Kit (Qiagen). The purified 1st PCR product was diluted to 0.1 ng µl^−1^ and used as a template for the 2nd PCR. Except for the multiplex reaction and multiple replications in the 1st PCR, all conditions and procedures for the 1st PCR, 2nd PCR and MiSeq sequencing were the same as used in the analysis of the mock communities.

### Data processing and taxonomic assignment of MiSeq reads

All data preprocessing and analysis of MiSeq raw reads were done using USEARCH v10.0.240^39^ by performing the following steps. (1) both forward and reverse reads were merged by aligning them using the *fastq_mergepairs* command. During this process, low-quality tail reads with a cut-off threshold set at a Phred score of 20 (=10^−2^ error rate), and those paired reads with too many differences (>5 positions) in the aligned region (ca. 70 bp) were discarded; (2) primer sequences were removed from those merged reads using the *fastx_truncate* command; (3) those reads without the primer sequences underwent quality filtering using the *fastq_filter* command to remove low quality reads with an expected error rate of >1% and too short reads of <120 bp; (4) the preprocessed reads were dereplicated using the *fastx_uniques* command and all singletons, doubletons and tripletons were removed from the subsequent analysis following the recommendation by ref.^39^; (5) the dereplicated reads were denoised using the *unoise3* command and, all putatively chimeric and erroneous sequences were separated from the subsequent operational taxonomic unit (OTU) assignment; and 6) finally all processed reads were assigned to OTU with a sequence identity of 98.5% (two nucleotide differences allowed) using the *usearch_global* command and the outputs were tabulated with read abundances. We processed the data of sequences reads as acceptable results when no reads had been detected from any blanks.

### Ethical statement

All experiments in this study were conducted without direct captures of live fish and were performed in accordance with the relevant guidelines and regulations. All experimental protocols were approved by Institutional guidelines for animal care of Nihon University.

## Results

### MiEel primers

**Figure 3:**
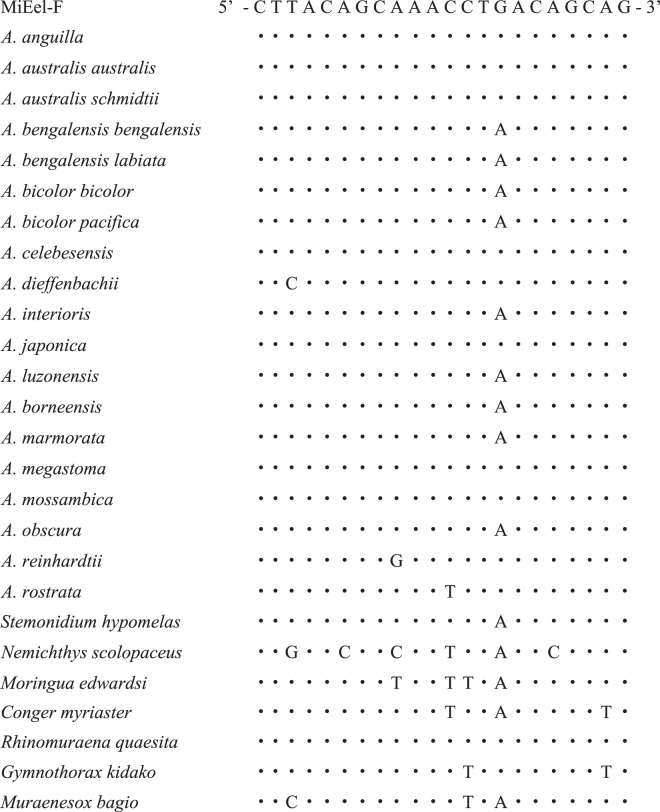
Nucleotide variations in the MiEel forward primers (MiEel-F) among 19 species/subspecies of *Anguilla* and other anguilliforms (*Stemonidium hypomelas*, *Nemichthys scolopaceus*, *Moringua edwardsi*, *Conger myriaster*, *Rhinomuraena quaesita*, *Gymnothorax kidako*, *Muraenesox bagio*). Dots indicate that nucleotides are identical to those of MiEel-F on the top line.

By visual examination of the aligned whole mitochondrial genomes from 19 species of *Anguilla*, two conserved regions that flank a variable region (167 bp) were located within the ATP6 gene. The new PCR primers for metabarcoding eDNA from the genus *Anguilla* were designed on the two conserved regions, and were named MiEel (Tables 1, 2). The MiEel-forward (MiEel-F) comprised 5′–CTTACAGCAAACCTGACAGCAG –3′, while the MiEel-reverse (MiEel-R) comprised 5′–TTGGTGTGCCATTATACGTTTTCTTG–3′ (universal primers, Table 1). The MiEel forward and reverse primers without adapter sequences consisted of 22 and 26 bases with T_m_ of 55.7 °C and 55.9 °C, and G/C content of 50% and 38% respectively. Of the 19 anguillid eels, 12 species had a single base pair mismatch in the MiEel forward primer (Fig. 3, Supplementary Fig. S1a). The 12 mismatches were unconventional T-G base pairing in 10 species and A-C base pairing in two species (Table 2). On the other hand, the reverse primer had identical sequence match with all reference sequences from *Anguilla* (Table 2, Supplementary Fig. S1b).

**Table 1:** A list of primers for the 1st PCR. MiEel primers (universal primers of *Anguilla* for eDNA metabarcoding).

Primers for the 1st-PCR	Sequence (5′-3′)
MiEel (forward)	ACACTCTTTCCCTACACGACGCTCTTCCGATCT NNNNNNCTTACAGCAAACCTGACAGCAG
MiEel (reverse)	GTGACTGGAGTTCAGACGTGTGCTCTTCCGATCT NNNNNNTTGGTGTGCCATTATACGTTTTCTTG

Underlines show universal primers for freshwater eels.

**Table 2:** Nucleotide sequences and base compositions of the MiEel forward primer (MiEel-F) and reverse primer (MiEel-R).

MiEel-F	5′-	C	T	T	A	C	A	G	C	A	A	A	C	C	T	G	A	C	A	G	C	A	G	−3′				
	A	0	0	0	19	0	19	0	0	18	19	19	0	0	0	9	19	0	19	0	0	19	0					
	C	19	0	1	0	19	0	0	19	0	0	0	18	19	0	0	0	19	0	0	19	0	0					
	G	0	0	0	0	0	0	19	0	1	0	0	0	0	0	10	0	0	0	19	0	0	19					
	T	0	19	18	0	0	0	0	0	0	0	0	1	0	19	0	0	0	0	0	0	0	0					
MiEel-R	5′-	T	T	G	G	T	G	T	G	C	C	A	T	T	A	T	A	C	G	T	T	T	T	C	T	T	G	−3′
	A	0	0	0	0	0	0	0	0	0	0	19	0	0	19	0	19	0	0	0	0	0	0	0	0	0	0	
	C	0	0	0	0	0	0	0	0	19	19	0	0	0	0	0	0	19	0	0	0	0	0	19	0	0	0	
	G	0	0	19	19	0	19	0	19	0	0	0	0	0	0	0	0	0	19	0	0	0	0	0	0	0	19	
	T	19	19	0	0	19	0	19	0	0	0	0	19	19	0	19	0	0	0	19	19	19	19	0	19	19	0

MiEel primers without adapter sequences were able to amplify each variable region of all 19 species and subspecies of the genus *Anguilla* using tissue-extracted DNA. The variable region of all *Anguilla* showed uncorrected pairwise distances from 0.6 to 13.2% with an average of 9% and nucleotide differences from one to 22 with an average of 14.

The sequence similarity between the MiEel primers and 19 other closely related anguilliform species appeared to be high (Supplementary Fig. S1c,d). The uncorrected pairwise distances for the variable regions between *Anguilla* and the anguilliforms ranged from 13.2 to 33.2% with an average 22.7% (Supplementary Fig. S2, Table S1). Therefore, MiEel primers would amplify eDNA from other anguilliforms, while the variable regions included sufficient nucleotide differences to distinguish *Anguilla* and the other anguilliforms.

The smallest nucleotide difference of one base pair was found between the two subspecies of *A. bengalensis*. Other subspecies groups (*A. australis* and *A. bicolor*) showed small intersubspecific variations with three base pair differences in the targeted ATP6 region. The neighbor-joining tree showed that the nucleotide differences in the ATP6 sequences amplified by MiEel primers were higher than those in the 12S rRNA regions amplified by MiFish primers with an average of 6 (range: 0–14) for *Anguilla* (Fig. 1), indicating that the MiEel primers had a higher taxonomic resolution to the genus.

### Species detection from mock communities

**Figure 4:**
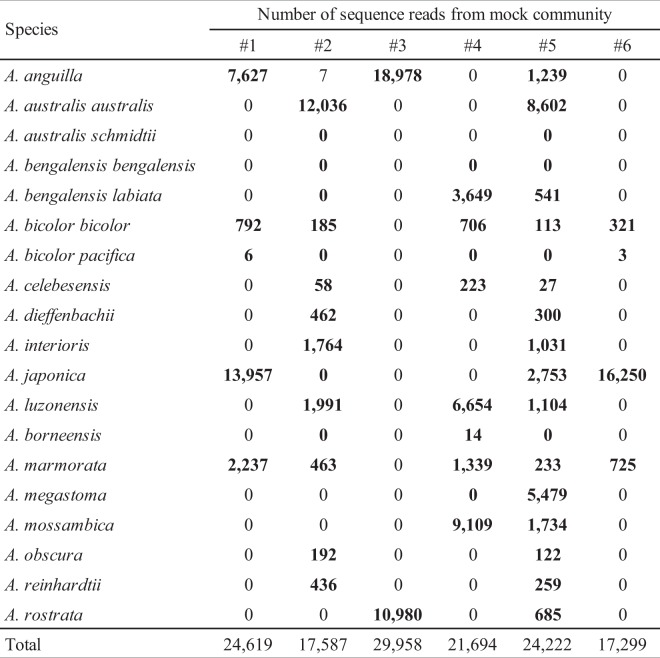
The number of the reads with identity of more than 98.5% from six mock communities. The bold values show that the species whose extracted DNA were added to mock communities.

Except for two subspecies (*A. australis schmidtii* and *A. bengalensis bengalensis*), the metabarcoding of six mock communities detected 17 species and subspecies of *Anguilla* (Fig. 4). The taxonomic assignment of the 17 detected species and subspecies corresponded with the species whose tissue-extracted DNA were added to each mock community. This demonstrated that the MiEel metabarcoding enabled the reads to be distinguished at the species level with identity of more than or equal to 98.5%. The number of sequence reads were variable among the species (Fig. 4). As the number of species whose tissue-extracted DNA were mixed into the same mock community increased, the sequence reads of each species tended to decrease (Fig. 4). *Anguilla bicolor pacifica* and *A. borneensis* were not found from the mock communities #2 and #5, and their overall detections were a few reads (3–14 reads) compared with other species (Fig. 4). Seven sequence reads were detected from *A. anguilla*, but its extracted DNA was not added to the mock community #2 (Fig. 4). The 1st PCR and the 2nd PCR blanks were negative.

### Species detection from eDNA samples

The MiEel eDNA metabarcoding detected the correct species living in the two aquarium tanks (Table 3). The eDNA from *A. bicolor bicolor* (224 reads) was detected from the tank that contained a single individual of that species. *Anguilla australis australis* (16,360 reads) was detected from the other tank containing *A. australis* sp. eel whose subspecies identification had been unclear.

**Table 3:** The number of reads with identify of more than 98.5% from eDNA samples from aquarium water in two tanks (Bibi, *A. bicolor bicolor*; Aust, *A. australis* sp.) and freshwater from the controlled pond and four rivers.

Species	Number of sequence reads from two aquarium tanks, controlled pond and four natural rivers							
	Bibi tank	Aust tank	Controlled pond	Ebitorigawa	Karasugawa	Ito-Nakagawa	Ito-Miyagawa estuary	Ito-Miyagawa upstream
*A. australis australis*	0	16,360	0	0	0	0	0	0
*A. australis schmidtii*	0	0	0	0	0	0	0	0
*A. bicolor bicolor*	224	0	0	0	0	0	0	0
*A. japonica*	0	0	8,014	8	479	702	299	466
Total	224	16,360	8,014	8	479	702	299	466

Out of 19 species and subspecies, four species detected from MiSeq sequencing were tabulated. Only *A. japonica* would be expected to be present the four rivers.

The MiEel analysis of the six freshwater samples from the controlled pond and natural rivers all detected eDNA from *A. japonica* without visual observation of the eels (Table 3). The more than 8000 sequence reads of *A. japonica* from the controlled pond was the highest number of reads among all freshwater samples. Only a modest eight reads were obtained from Ebitorigawa River, and more than 290 reads were detected from the other three rivers (Table 3). All filtration, extraction, the 1st PCR and the 2nd PCR blanks yielded no reads of the genus *Anguilla*, providing no indication of contamination.

## Discussion

### Utility of eDNA metabarcoding with MiEel primers

The development of the MiEel primers enabled sequence reads of freshwater eels to be assigned at the species level. With the newly designed MiEel primers without adapter sequences, variable sequences (167 bp) within the ATP6 gene were confirmed using the tissue-derived DNA extracts from 19 *Anguilla* species/subspecies. The variable sequences could be used to distinguish each species of *Anguilla*. The similarity of sequences between the MiEel primers and the ATP6 sequences of some species of anguilliforms including *Anguilla* indicated that there is a possibility that the primers could amplify eDNA from other anguilliforms (Supplementary Fig. S1c,d). However, there would be no risk of incorrect taxonomic assignments through misidentification of sequence reads because the 167 bp of ATP6 sequences that would be amplified by the MiEel primers included sufficient nucleotide differences between *Anguilla* and other anguilliforms (Supplementary Fig. S2, Table S1). Therefore, the MiEel primer should work well for eDNA metabarcoding to *Anguilla*.

The MiEel metabarcoding with six mock communities containing 2–19 *Anguilla* species tissue DNA extracts demonstrated a simultaneous PCR amplification and taxonomic assignment at the species level, but failed to detect two subspecies *A. australis schmidtii* and *A. bengalensis bengalensis* (Fig. 4). In addition, eDNA of some freshwater eels were successfully detected from two aquarium tanks, the controlled pond, and four natural rivers (Table 3). Therefore, the eDNA metabarcoding with the MiEel primers provided information on the presence or absence of some *Anguilla* species without capturing them, which indicated it can be a useful approach to estimate their distribution in freshwater and marine habitats.

The MiEel metabarcoding would be particularly effective for documenting species distributions in aquatic environments where many *Anguilla* species might occur sympatrically. For example, eight species probably occur within and near the Indonesian archipelago that include *A. bengalensis*, *A. bicolor*, *A. borneensis*, *A. celebesensis*, *A. interioris*, *A. marmorata*, *A. megastoma*, and *A. obscura*^40^. There are also six anguillid species that live and spawn in the western South Pacific region^41,42^. Species identification of those *Anguilla* species still remains difficult because morphological traits of their larvae partially or completely overlap among some species and genetic identification is required^43,44^, and the same problem exists for some of their adults^45^. Genetic species identification of collected specimens of *Anguilla* has been conducted using a portion of 16S rRNA and cyt *b* genes^43,44,46^. Even if genetic identification is used, intensive efforts are still required to collect the eels or larvae to identify the distribution and possible spawning areas. In contrast, the MiEel metabarcoding allowed simultaneous detection of freshwater eels at the species level from water samples without much sampling effort and expertise in taxonomy (Fig. 4, Table 3). If the aim is to examine the presence/absence of several species of *Anguilla* that can be sympatrically distributed in aquatic environments like in the Indonesian archipelago, on islands of the western South Pacific and in offshore spawning areas, eDNA metabarcoding with the MiEel primers will provide fast and efficient insight into the distribution. The MiEel metabarcoding is expected to work as a powerful supplement to the conventional sampling methods for natural resource management and ecological studies of freshwater eels.

### Taxonomic assignment to freshwater eels

Our study found, however, that the discrimination of the three subspecies pairs *A. australis*, *A. bengalensis*, *A. bicolor* may be ambiguous when using the eDNA metabarcoding with MiEel primers. The two subspecies of *A. bengalensis* had the smallest nucleotide differences of one base among all species/subspecies pairs, which is followed by the two subspecies pairs of *A. australis* and *A. bicolor* with three nucleotide differences (Fig. 1). Such small differences may pose serious limits for subspecies assignment because of erroneous sequence reads from an NGS platform. Although a denoising process was introduced in the present analysis, there were still erroneous sequences, which will introduce both false positives and negatives^39^. Fortunately, the three subspecies likely do not usually overlap in the geography of their freshwater habitats^47^.

The limitation for distinguishing anguillid subspecies suggested though, the taxonomic assignment of sequence reads to the freshwater eels can still be improved. Species identification using eDNA metabarcoding crucially depends on correct and reliable reference sequence database^9,14,48,49^, so the database must consist of DNA sequences from properly vouchered and correctly identified species. In this study, reference sequences were derived from 167 bp of ATP6 sequences from each species and subspecies of *Anguilla* that were morphologically and genetically identified (19 sequences; see Methods). The 167 bp sequences of 13 *Anguilla* species were identical with those registered previously in DDBJ by Minegishi *et al*.^28,29^, but there was one mismatch for *A. reinhardtii*, *A. australis schmidtii* and *A. dieffenbachii*, and were two mismatches for *A. celebesensis* and *A. bicolor pacifica*. These mismatches were unable to be determined as intraspecific variations because of few reliable ATP6 sequences of *Anguilla* being available in public databases. The improvement of the sequence assignment requires an increase of ATP6 sequence data verified by a concerted effort between taxonomists and molecular systematists.

### False positive and false negative

A false positive (species is detected where it is not present) and false negative (species is not detected where it is present) have been frequently discussed as an important issue in eDNA studies^9,14,49^. For example, seven sequence reads of *A. anguilla* were detected from the mock community #2 although the tissue-derived DNA of that species was not mixed into it (Fig. 4), indicating a false positive occurred. Laboratory contamination is especially serious because of frequent use of PCR generating billions of DNA copies, which can easily spread throughout a laboratory^49^. Moreover, handling PCR products and evaporation of these may result in DNA invasion into the surrounding air and subsequent formation of DNA aerosols, which cause a risk of contamination^50,51^. The tissue DNA extraction of freshwater eels and the library preparation for the MiEel metabarcoding using the mock communities was conducted in the same work space. Some aerosolized DNA that produce false positives might have been present in the work space when analyzing the mock communities. Since the 1st PCR and the 2nd PCR blanks turned out negative, the source of low sequence reads from *A. anguilla* most likely was aerosolized DNA being in the work space when the six mock communities were created.

In contrast, *A. australis schmidtii*, *A. bengalensis bengalensis*, *A. bicolor pacifica* and *A. borneensis* were usually not detected from the mock communities #2, #4 or #5 containing ≥8 species and subspecies, which showed the occurrence of false negatives despite their tissue DNA being added to the mock communities. PCR bias derived from primer-template mismatches is a possible factor causing imperfect detections^9^. Importantly, MiEel primers were able to amplify each variable region for all freshwater eels using tissue extracted DNA when analyzed separately. This implied that MiEel metabarcoding should be able to simultaneously detect multiple anguillid eels from a single water sample. However, amplification efficiencies for all DNA templates in the mock communities containing many species (a large number of competing templates) are not always equal because 12 anguillid eels including *A. bengalensis bengalensis*, *A. bicolor pacifica* and *A. borneensis* have one mismatch with the MiEel forward primer (Fig. 3). Because there are problems with the stochasticity of individual PCR reactions and PCR bias derived from primer-template mismatches, the eDNA metabarcoding needs some methodological improvements to mitigate PCR dropouts in cases with many templates in a single sample. Increasing the number of PCR replications and pooling them were recommended to reduce the risk of false negatives in eDNA metabarcoding studies^9,38,52^. However, it is unlikely that a single water sample will include eight or more anguillid eels in natural environments.

To avoid the false positive and false negative detections, which were in fact observed in the analysis of the mock communities, the eDNA samples were analyzed with some optimizations to strictly monitor contamination and to increase a detection probability. All blanks (filtration, extraction, the 1st PCR and the 2nd PCR) were processed in parallel to the analysis of eDNA samples, and then produced negative results. Thus, eDNA samples may not have been contaminated with any DNA from freshwater eels during filtration, extraction, the 1st PCR and the 2nd PCR. Unlike the unexpected detection of *A. anguilla* from the mock community #2 (Fig. 4), the MiEel metabarcoding using the eDNA samples yielded no reads of irrelevant *Anguilla* species (Table 3). This implied that the strict clean-lab procedures (e.g. separation of the working place) worked well for safeguarding contamination. Moreover, eight PCR replicates per one of each eDNA extract were subjected to the 1st PCR. To reduce the rate of false negatives, the eight PCR replicates should be performed if detection probability is low^38^. The on-site filtration method at water sampling sites using the filter cartridges and the syringe is also ideal to minimize false negatives because this method will provide more intact eDNA^37^. Without those optimal strategies to minimize false positive and false negative, it was impossible to recover low abundant sequences such as the reads of Ebitorigawa River, in which *A. japonica* is expected to be present, from the field eDNA samples (Table 3). The sequence reads of *A. japonica* detected from the Ebitorigawa River were only a modest eight reads, but verified that the eDNA metabarcoding using the MiEel reflected the species distribution of *Anguilla* there because no reads were detected from any blanks. This result emphasized the importance of strict and optimal protocols to detect target species with confidence, especially when the application of the eDNA metabarcoding in the field.

## Supplementary information

Supplementary Information

## Data Availability

Raw reads of the mock communities and eDNA samples from the MiSeq sequencing are available from the DDBJ Sequence Reads Archive (Accession Number DRA005271 and DRA006504).
