# Supplementary material for: New PCR primers for metabarcoding environmental DNA from freshwater eels, genus Anguilla
Source: Sci Rep. 2019 May 28;9:7977. doi: 10.1038/s41598-019-44402-0 (PMC6538671; doi:10.1038/s41598-019-44402-0)
Supplement: Supplementary file 1 — Supplementary Information [file 41598_2019_44402_MOESM1_ESM.docx]

**Supplementary Information**

**New PCR primers for metabarcoding environmental DNA from freshwater eels, genus *Anguilla***

Aya Takeuchi^1,*^, Tetsuya Sado^2^, Ryo O. Gotoh^2^, Shun Watanabe^3^, Katsumi Tsukamoto^1,4^ & Masaki Miya^2,*^

^1^Graduate School of Bioresource Sciences, Nihon University, 1866 Kameino, Fujisawa, Kanagawa, 252-0880, Japan; ^2^Department of Ecology and Environmental Sciences, Natural History Museum and Institute, 955-2 Aoba-cho, Chuo, Chiba, 260-8682, Japan; ^3^Department of Fisheries, Faculty of Agriculture, Kindai University, 3327-204 Nakamachi, Nara, 631-8505, Japan; ^4^Present address: Department of Aquatic Bioscience, Graduate School of Agricultural and Life Sciences, The University of Tokyo, 1-1-1 Yayoi, Bunkyo, Tokyo, 113-8657, Japan

*Corresponding author:

Aya Takeuchi

Address: Graduate School of Bioresource Sciences, Nihon University, 1866 Kameino, Fujisawa, Kanagawa, 252-0880, Japan.

Email: ka030ak@yahoo.co.jp

Masaki Miya

Address: Department of Ecology and Environmental Sciences, Natural History Museum and Institute, 955-2 Aoba-cho, Chuo, Chiba, 260-8682, Japan.

Email: miya@chiba-muse.or.jp

**Contents**

**Figure S1**. Sequence logo plots between the MiEel primers and the genus *Anguilla*, and 19 species of the Anguilliform.

**Figure S2**. Neighbor-joining tree of 19 *Anguilla* and 19 other anguilliform species based on the variable sequences of 167 bp within the ATP6 region.

**Table S1**. Pairwise distances between *Anguilla* and 19 species of the Anguilliform.

**Supplementary References**

1. Crooks, G. E., Hon, G., Chandonia, J. M. & Brenner, S. E. WebLogo: A sequence logo generator. *Genome Res.* **14**, 1188–1190 (2004).
2. Tamura, K., Stecher, G., Peterson, D., Filipski, A. & Kumar, S. MEGA6: molecular evolutionary genetics analysis version 6.0. *Mol Biol Evol*. **30**, 2725−2729 (2013).

**Figure S1**. Sequence logo plots using WebLogo^1^ between the MiEel-F/R primers and the genus *Anguilla* (a, b), and 19 species of the Anguilliforms (c, d).

**Figure S2**. Neighbor-joining tree of 19 *Anguilla* and 19 other anguilliforms based on the variable sequences of 167 bp within the ATP6 region. Distance was calculated using the Kimura two parameter model with all sequences containing gaps being eliminated, and numerals beside the internal branches are bootstrap probabilities of 300 replicates. This analysis of phylogenetic tree was conducted in MEGA6^2^.

**Table S1.** Pairwise distances between *Anguilla* and 19 species of the Anguilliform. An analysis was conducted using the Kimura two parameter model in MEGA6^2^.

| Species name | Species name | Distance |
| --- | --- | --- |
| *Anguilla_australis_australis* | *Stemonidium_hypomelas* | 0.132 |
| *Anguilla_australis_schmidtii* | *Stemonidium_hypomelas* | 0.139 |
| *Anguilla_bicolor_bicolor* | *Stemonidium_hypomelas* | 0.125 |
| *Anguilla_bicolor_pacifica* | *Stemonidium_hypomelas* | 0.125 |
| *Anguilla_bengalensis_labiata* | *Stemonidium_hypomelas* | 0.147 |
| *Anguilla_bengalensis_bengalensis* | *Stemonidium_hypomelas* | 0.139 |
| *Anguilla_anguilla* | *Stemonidium_hypomelas* | 0.147 |
| *Anguilla_borneensis* | *Stemonidium_hypomelas* | 0.154 |
| *Anguilla_celebesensis* | *Stemonidium_hypomelas* | 0.139 |
| *Anguilla_dieffenbachii* | *Stemonidium_hypomelas* | 0.132 |
| *Anguilla_interioris* | *Stemonidium_hypomelas* | 0.126 |
| *Anguilla_japonica* | *Stemonidium_hypomelas* | 0.156 |
| *Anguilla_marmorata* | *Stemonidium_hypomelas* | 0.125 |
| *Anguilla_megastoma* | *Stemonidium_hypomelas* | 0.148 |
| *Anguilla_mossambica* | *Stemonidium_hypomelas* | 0.139 |
| *Anguilla_obscura* | *Stemonidium_hypomelas* | 0.103 |
| *Anguilla_reinhardtii* | *Stemonidium_hypomelas* | 0.156 |
| *Anguilla_rostrata* | *Stemonidium_hypomelas* | 0.163 |
| *Anguilla_luzonensis* | *Stemonidium_hypomelas* | 0.126 |
| *Anguilla_australis_australis* | *Moringua_edwardsi* | 0.277 |
| *Anguilla_australis_schmidtii* | *Moringua_edwardsi* | 0.268 |
| *Anguilla_bicolor_bicolor* | *Moringua_edwardsi* | 0.249 |
| *Anguilla_bicolor_pacifica* | *Moringua_edwardsi* | 0.249 |
| *Anguilla_bengalensis_labiata* | *Moringua_edwardsi* | 0.250 |
| *Anguilla_bengalensis_bengalensis* | *Moringua_edwardsi* | 0.250 |
| *Anguilla_anguilla* | *Moringua_edwardsi* | 0.344 |
| *Anguilla_borneensis* | *Moringua_edwardsi* | 0.296 |
| *Anguilla_celebesensis* | *Moringua_edwardsi* | 0.268 |
| *Anguilla_dieffenbachii* | *Moringua_edwardsi* | 0.258 |
| *Anguilla_interioris* | *Moringua_edwardsi* | 0.267 |
| *Anguilla_japonica* | *Moringua_edwardsi* | 0.286 |

**Table S1.** (Continued)

| *Anguilla_marmorata* | *Moringua_edwardsi* | 0.248 |
| --- | --- | --- |
| *Anguilla_megastoma* | *Moringua_edwardsi* | 0.251 |
| *Anguilla_mossambica* | *Moringua_edwardsi* | 0.277 |
| *Anguilla_obscura* | *Moringua_edwardsi* | 0.267 |
| *Anguilla_reinhardtii* | *Moringua_edwardsi* | 0.268 |
| *Anguilla_rostrata* | *Moringua_edwardsi* | 0.315 |
| *Anguilla_luzonensis* | *Moringua_edwardsi* | 0.276 |
| *Anguilla_australis_australis* | *Kaupichthys_hyoproroides* | 0.286 |
| *Anguilla_australis_schmidtii* | *Kaupichthys_hyoproroides* | 0.297 |
| *Anguilla_bicolor_bicolor* | *Kaupichthys_hyoproroides* | 0.326 |
| *Anguilla_bicolor_pacifica* | *Kaupichthys_hyoproroides* | 0.337 |
| *Anguilla_bengalensis_labiata* | *Kaupichthys_hyoproroides* | 0.357 |
| *Anguilla_bengalensis_bengalensis* | *Kaupichthys_hyoproroides* | 0.346 |
| *Anguilla_anguilla* | *Kaupichthys_hyoproroides* | 0.326 |
| *Anguilla_borneensis* | *Kaupichthys_hyoproroides* | 0.326 |
| *Anguilla_celebesensis* | *Kaupichthys_hyoproroides* | 0.307 |
| *Anguilla_dieffenbachii* | *Kaupichthys_hyoproroides* | 0.286 |
| *Anguilla_interioris* | *Kaupichthys_hyoproroides* | 0.318 |
| *Anguilla_japonica* | *Kaupichthys_hyoproroides* | 0.316 |
| *Anguilla_marmorata* | *Kaupichthys_hyoproroides* | 0.335 |
| *Anguilla_megastoma* | *Kaupichthys_hyoproroides* | 0.306 |
| *Anguilla_mossambica* | *Kaupichthys_hyoproroides* | 0.294 |
| *Anguilla_obscura* | *Kaupichthys_hyoproroides* | 0.359 |
| *Anguilla_reinhardtii* | *Kaupichthys_hyoproroides* | 0.297 |
| *Anguilla_rostrata* | *Kaupichthys_hyoproroides* | 0.276 |
| *Anguilla_luzonensis* | *Kaupichthys_hyoproroides* | 0.372 |
| *Anguilla_australis_australis* | *Robinsia_catherinae* | 0.233 |
| *Anguilla_australis_schmidtii* | *Robinsia_catherinae* | 0.217 |
| *Anguilla_bicolor_bicolor* | *Robinsia_catherinae* | 0.233 |
| *Anguilla_bicolor_pacifica* | *Robinsia_catherinae* | 0.216 |
| *Anguilla_bengalensis_labiata* | *Robinsia_catherinae* | 0.241 |
| *Anguilla_bengalensis_bengalensis* | *Robinsia_catherinae* | 0.232 |
| *Anguilla_anguilla* | *Robinsia_catherinae* | 0.218 |

**Table S1.** (Continued)

| *Anguilla_borneensis* | *Robinsia_catherinae* | 0.216 |
| --- | --- | --- |
| *Anguilla_celebesensis* | *Robinsia_catherinae* | 0.250 |
| *Anguilla_dieffenbachii* | *Robinsia_catherinae* | 0.226 |
| *Anguilla_interioris* | *Robinsia_catherinae* | 0.232 |
| *Anguilla_japonica* | *Robinsia_catherinae* | 0.199 |
| *Anguilla_marmorata* | *Robinsia_catherinae* | 0.278 |
| *Anguilla_megastoma* | *Robinsia_catherinae* | 0.270 |
| *Anguilla_mossambica* | *Robinsia_catherinae* | 0.218 |
| *Anguilla_obscura* | *Robinsia_catherinae* | 0.251 |
| *Anguilla_reinhardtii* | *Robinsia_catherinae* | 0.243 |
| *Anguilla_rostrata* | *Robinsia_catherinae* | 0.185 |
| *Anguilla_luzonensis* | *Robinsia_catherinae* | 0.268 |
| *Anguilla_australis_australis* | *Gymnothorax_kidako* | 0.330 |
| *Anguilla_australis_schmidtii* | *Gymnothorax_kidako* | 0.301 |
| *Anguilla_bicolor_bicolor* | *Gymnothorax_kidako* | 0.308 |
| *Anguilla_bicolor_pacifica* | *Gymnothorax_kidako* | 0.308 |
| *Anguilla_bengalensis_labiata* | *Gymnothorax_kidako* | 0.317 |
| *Anguilla_bengalensis_bengalensis* | *Gymnothorax_kidako* | 0.317 |
| *Anguilla_anguilla* | *Gymnothorax_kidako* | 0.281 |
| *Anguilla_borneensis* | *Gymnothorax_kidako* | 0.318 |
| *Anguilla_celebesensis* | *Gymnothorax_kidako* | 0.292 |
| *Anguilla_dieffenbachii* | *Gymnothorax_kidako* | 0.318 |
| *Anguilla_interioris* | *Gymnothorax_kidako* | 0.300 |
| *Anguilla_japonica* | *Gymnothorax_kidako* | 0.246 |
| *Anguilla_marmorata* | *Gymnothorax_kidako* | 0.337 |
| *Anguilla_megastoma* | *Gymnothorax_kidako* | 0.318 |
| *Anguilla_mossambica* | *Gymnothorax_kidako* | 0.348 |
| *Anguilla_obscura* | *Gymnothorax_kidako* | 0.318 |
| *Anguilla_reinhardtii* | *Gymnothorax_kidako* | 0.319 |
| *Anguilla_rostrata* | *Gymnothorax_kidako* | 0.289 |
| *Anguilla_luzonensis* | *Gymnothorax_kidako* | 0.300 |
| *Anguilla_australis_australis* | *Rhinomuraena_quaesita* | 0.300 |
| *Anguilla_australis_schmidtii* | *Rhinomuraena_quaesita* | 0.308 |

**Table S1.** (Continued)

| *Anguilla_bicolor_bicolor* | *Rhinomuraena_quaesita* | 0.292 |
| --- | --- | --- |
| *Anguilla_bicolor_pacifica* | *Rhinomuraena_quaesita* | 0.292 |
| *Anguilla_bengalensis_labiata* | *Rhinomuraena_quaesita* | 0.293 |
| *Anguilla_bengalensis_bengalensis* | *Rhinomuraena_quaesita* | 0.293 |
| *Anguilla_anguilla* | *Rhinomuraena_quaesita* | 0.289 |
| *Anguilla_borneensis* | *Rhinomuraena_quaesita* | 0.317 |
| *Anguilla_celebesensis* | *Rhinomuraena_quaesita* | 0.308 |
| *Anguilla_dieffenbachii* | *Rhinomuraena_quaesita* | 0.298 |
| *Anguilla_interioris* | *Rhinomuraena_quaesita* | 0.283 |
| *Anguilla_japonica* | *Rhinomuraena_quaesita* | 0.290 |
| *Anguilla_marmorata* | *Rhinomuraena_quaesita* | 0.312 |
| *Anguilla_megastoma* | *Rhinomuraena_quaesita* | 0.301 |
| *Anguilla_mossambica* | *Rhinomuraena_quaesita* | 0.329 |
| *Anguilla_obscura* | *Rhinomuraena_quaesita* | 0.301 |
| *Anguilla_reinhardtii* | *Rhinomuraena_quaesita* | 0.282 |
| *Anguilla_rostrata* | *Rhinomuraena_quaesita* | 0.254 |
| *Anguilla_luzonensis* | *Rhinomuraena_quaesita* | 0.343 |
| *Anguilla_australis_australis* | *Ilyophis_brunneus* | 0.276 |
| *Anguilla_australis_schmidtii* | *Ilyophis_brunneus* | 0.275 |
| *Anguilla_bicolor_bicolor* | *Ilyophis_brunneus* | 0.315 |
| *Anguilla_bicolor_pacifica* | *Ilyophis_brunneus* | 0.295 |
| *Anguilla_bengalensis_labiata* | *Ilyophis_brunneus* | 0.294 |
| *Anguilla_bengalensis_bengalensis* | *Ilyophis_brunneus* | 0.284 |
| *Anguilla_anguilla* | *Ilyophis_brunneus* | 0.274 |
| *Anguilla_borneensis* | *Ilyophis_brunneus* | 0.267 |
| *Anguilla_celebesensis* | *Ilyophis_brunneus* | 0.267 |
| *Anguilla_dieffenbachii* | *Ilyophis_brunneus* | 0.295 |
| *Anguilla_interioris* | *Ilyophis_brunneus* | 0.249 |
| *Anguilla_japonica* | *Ilyophis_brunneus* | 0.259 |
| *Anguilla_marmorata* | *Ilyophis_brunneus* | 0.323 |
| *Anguilla_megastoma* | *Ilyophis_brunneus* | 0.248 |
| *Anguilla_mossambica* | *Ilyophis_brunneus* | 0.285 |
| *Anguilla_obscura* | *Ilyophis_brunneus* | 0.276 |

**Table S1.** (Continued)

| *Anguilla_reinhardtii* | *Ilyophis_brunneus* | 0.326 |
| --- | --- | --- |
| *Anguilla_rostrata* | *Ilyophis_brunneus* | 0.264 |
| *Anguilla_luzonensis* | *Ilyophis_brunneus* | 0.316 |
| *Anguilla_australis_australis* | *Synaphobranchus_kaupii* | 0.285 |
| *Anguilla_australis_schmidtii* | *Synaphobranchus_kaupii* | 0.284 |
| *Anguilla_bicolor_bicolor* | *Synaphobranchus_kaupii* | 0.305 |
| *Anguilla_bicolor_pacifica* | *Synaphobranchus_kaupii* | 0.305 |
| *Anguilla_bengalensis_labiata* | *Synaphobranchus_kaupii* | 0.303 |
| *Anguilla_bengalensis_bengalensis* | *Synaphobranchus_kaupii* | 0.294 |
| *Anguilla_anguilla* | *Synaphobranchus_kaupii* | 0.302 |
| *Anguilla_borneensis* | *Synaphobranchus_kaupii* | 0.295 |
| *Anguilla_celebesensis* | *Synaphobranchus_kaupii* | 0.277 |
| *Anguilla_dieffenbachii* | *Synaphobranchus_kaupii* | 0.305 |
| *Anguilla_interioris* | *Synaphobranchus_kaupii* | 0.258 |
| *Anguilla_japonica* | *Synaphobranchus_kaupii* | 0.250 |
| *Anguilla_marmorata* | *Synaphobranchus_kaupii* | 0.334 |
| *Anguilla_megastoma* | *Synaphobranchus_kaupii* | 0.257 |
| *Anguilla_mossambica* | *Synaphobranchus_kaupii* | 0.295 |
| *Anguilla_obscura* | *Synaphobranchus_kaupii* | 0.285 |
| *Anguilla_reinhardtii* | *Synaphobranchus_kaupii* | 0.316 |
| *Anguilla_rostrata* | *Synaphobranchus_kaupii* | 0.292 |
| *Anguilla_luzonensis* | *Synaphobranchus_kaupii* | 0.326 |
| *Anguilla_australis_australis* | *Simenchelys_parasitica* | 0.255 |
| *Anguilla_australis_schmidtii* | *Simenchelys_parasitica* | 0.230 |
| *Anguilla_bicolor_bicolor* | *Simenchelys_parasitica* | 0.255 |
| *Anguilla_bicolor_pacifica* | *Simenchelys_parasitica* | 0.255 |
| *Anguilla_bengalensis_labiata* | *Simenchelys_parasitica* | 0.264 |
| *Anguilla_bengalensis_bengalensis* | *Simenchelys_parasitica* | 0.273 |
| *Anguilla_anguilla* | *Simenchelys_parasitica* | 0.273 |
| *Anguilla_borneensis* | *Simenchelys_parasitica* | 0.282 |
| *Anguilla_celebesensis* | *Simenchelys_parasitica* | 0.257 |
| *Anguilla_dieffenbachii* | *Simenchelys_parasitica* | 0.238 |
| *Anguilla_interioris* | *Simenchelys_parasitica* | 0.256 |

**Table S1.** (Continued)

| *Anguilla_japonica* | *Simenchelys_parasitica* | 0.284 |
| --- | --- | --- |
| *Anguilla_marmorata* | *Simenchelys_parasitica* | 0.282 |
| *Anguilla_megastoma* | *Simenchelys_parasitica* | 0.273 |
| *Anguilla_mossambica* | *Simenchelys_parasitica* | 0.264 |
| *Anguilla_obscura* | *Simenchelys_parasitica* | 0.255 |
| *Anguilla_reinhardtii* | *Simenchelys_parasitica* | 0.283 |
| *Anguilla_rostrata* | *Simenchelys_parasitica* | 0.263 |
| *Anguilla_luzonensis* | *Simenchelys_parasitica* | 0.302 |
| *Anguilla_australis_australis* | *Ophisurus_macrorhynchos* | 0.295 |
| *Anguilla_australis_schmidtii* | *Ophisurus_macrorhynchos* | 0.267 |
| *Anguilla_bicolor_bicolor* | *Ophisurus_macrorhynchos* | 0.312 |
| *Anguilla_bicolor_pacifica* | *Ophisurus_macrorhynchos* | 0.312 |
| *Anguilla_bengalensis_labiata* | *Ophisurus_macrorhynchos* | 0.321 |
| *Anguilla_bengalensis_bengalensis* | *Ophisurus_macrorhynchos* | 0.321 |
| *Anguilla_anguilla* | *Ophisurus_macrorhynchos* | 0.274 |
| *Anguilla_borneensis* | *Ophisurus_macrorhynchos* | 0.302 |
| *Anguilla_celebesensis* | *Ophisurus_macrorhynchos* | 0.294 |
| *Anguilla_dieffenbachii* | *Ophisurus_macrorhynchos* | 0.285 |
| *Anguilla_interioris* | *Ophisurus_macrorhynchos* | 0.294 |
| *Anguilla_japonica* | *Ophisurus_macrorhynchos* | 0.285 |
| *Anguilla_marmorata* | *Ophisurus_macrorhynchos* | 0.311 |
| *Anguilla_megastoma* | *Ophisurus_macrorhynchos* | 0.322 |
| *Anguilla_mossambica* | *Ophisurus_macrorhynchos* | 0.305 |
| *Anguilla_obscura* | *Ophisurus_macrorhynchos* | 0.322 |
| *Anguilla_reinhardtii* | *Ophisurus_macrorhynchos* | 0.323 |
| *Anguilla_rostrata* | *Ophisurus_macrorhynchos* | 0.264 |
| *Anguilla_luzonensis* | *Ophisurus_macrorhynchos* | 0.323 |
| *Anguilla_australis_australis* | *Nessorhamphus_ingolfianus* | 0.275 |
| *Anguilla_australis_schmidtii* | *Nessorhamphus_ingolfianus* | 0.267 |
| *Anguilla_bicolor_bicolor* | *Nessorhamphus_ingolfianus* | 0.296 |
| *Anguilla_bicolor_pacifica* | *Nessorhamphus_ingolfianus* | 0.296 |
| *Anguilla_bengalensis_labiata* | *Nessorhamphus_ingolfianus* | 0.346 |
| *Anguilla_bengalensis_bengalensis* | *Nessorhamphus_ingolfianus* | 0.335 |

**Table S1.** (Continued)

| *Anguilla_anguilla* | *Nessorhamphus_ingolfianus* | 0.257 |
| --- | --- | --- |
| *Anguilla_borneensis* | *Nessorhamphus_ingolfianus* | 0.316 |
| *Anguilla_celebesensis* | *Nessorhamphus_ingolfianus* | 0.288 |
| *Anguilla_dieffenbachii* | *Nessorhamphus_ingolfianus* | 0.323 |
| *Anguilla_interioris* | *Nessorhamphus_ingolfianus* | 0.315 |
| *Anguilla_japonica* | *Nessorhamphus_ingolfianus* | 0.337 |
| *Anguilla_marmorata* | *Nessorhamphus_ingolfianus* | 0.305 |
| *Anguilla_megastoma* | *Nessorhamphus_ingolfianus* | 0.306 |
| *Anguilla_mossambica* | *Nessorhamphus_ingolfianus* | 0.286 |
| *Anguilla_obscura* | *Nessorhamphus_ingolfianus* | 0.326 |
| *Anguilla_reinhardtii* | *Nessorhamphus_ingolfianus* | 0.307 |
| *Anguilla_rostrata* | *Nessorhamphus_ingolfianus* | 0.274 |
| *Anguilla_luzonensis* | *Nessorhamphus_ingolfianus* | 0.335 |
| *Anguilla_australis_australis* | *Muraenesox_bagio* | 0.275 |
| *Anguilla_australis_schmidtii* | *Muraenesox_bagio* | 0.276 |
| *Anguilla_bicolor_bicolor* | *Muraenesox_bagio* | 0.273 |
| *Anguilla_bicolor_pacifica* | *Muraenesox_bagio* | 0.282 |
| *Anguilla_bengalensis_labiata* | *Muraenesox_bagio* | 0.293 |
| *Anguilla_bengalensis_bengalensis* | *Muraenesox_bagio* | 0.293 |
| *Anguilla_anguilla* | *Muraenesox_bagio* | 0.311 |
| *Anguilla_borneensis* | *Muraenesox_bagio* | 0.275 |
| *Anguilla_celebesensis* | *Muraenesox_bagio* | 0.276 |
| *Anguilla_dieffenbachii* | *Muraenesox_bagio* | 0.294 |
| *Anguilla_interioris* | *Muraenesox_bagio* | 0.265 |
| *Anguilla_japonica* | *Muraenesox_bagio* | 0.303 |
| *Anguilla_marmorata* | *Muraenesox_bagio* | 0.310 |
| *Anguilla_megastoma* | *Muraenesox_bagio* | 0.284 |
| *Anguilla_mossambica* | *Muraenesox_bagio* | 0.277 |
| *Anguilla_obscura* | *Muraenesox_bagio* | 0.282 |
| *Anguilla_reinhardtii* | *Muraenesox_bagio* | 0.322 |
| *Anguilla_rostrata* | *Muraenesox_bagio* | 0.310 |
| *Anguilla_luzonensis* | *Muraenesox_bagio* | 0.312 |
| *Anguilla_australis_australis* | *Nemichthys_scolopaceus* | 0.268 |

**Table S1.** (Continued)

| *Anguilla_australis_schmidtii* | *Nemichthys_scolopaceus* | 0.286 |
| --- | --- | --- |
| *Anguilla_bicolor_bicolor* | *Nemichthys_scolopaceus* | 0.315 |
| *Anguilla_bicolor_pacifica* | *Nemichthys_scolopaceus* | 0.305 |
| *Anguilla_bengalensis_labiata* | *Nemichthys_scolopaceus* | 0.294 |
| *Anguilla_bengalensis_bengalensis* | *Nemichthys_scolopaceus* | 0.284 |
| *Anguilla_anguilla* | *Nemichthys_scolopaceus* | 0.295 |
| *Anguilla_borneensis* | *Nemichthys_scolopaceus* | 0.268 |
| *Anguilla_celebesensis* | *Nemichthys_scolopaceus* | 0.258 |
| *Anguilla_dieffenbachii* | *Nemichthys_scolopaceus* | 0.297 |
| *Anguilla_interioris* | *Nemichthys_scolopaceus* | 0.286 |
| *Anguilla_japonica* | *Nemichthys_scolopaceus* | 0.259 |
| *Anguilla_marmorata* | *Nemichthys_scolopaceus* | 0.303 |
| *Anguilla_megastoma* | *Nemichthys_scolopaceus* | 0.267 |
| *Anguilla_mossambica* | *Nemichthys_scolopaceus* | 0.274 |
| *Anguilla_obscura* | *Nemichthys_scolopaceus* | 0.315 |
| *Anguilla_reinhardtii* | *Nemichthys_scolopaceus* | 0.326 |
| *Anguilla_rostrata* | *Nemichthys_scolopaceus* | 0.266 |
| *Anguilla_luzonensis* | *Nemichthys_scolopaceus* | 0.306 |
| *Anguilla_australis_australis* | *Avocettina_infans* | 0.261 |
| *Anguilla_australis_schmidtii* | *Avocettina_infans* | 0.244 |
| *Anguilla_bicolor_bicolor* | *Avocettina_infans* | 0.271 |
| *Anguilla_bicolor_pacifica* | *Avocettina_infans* | 0.271 |
| *Anguilla_bengalensis_labiata* | *Avocettina_infans* | 0.270 |
| *Anguilla_bengalensis_bengalensis* | *Avocettina_infans* | 0.260 |
| *Anguilla_anguilla* | *Avocettina_infans* | 0.245 |
| *Anguilla_borneensis* | *Avocettina_infans* | 0.271 |
| *Anguilla_celebesensis* | *Avocettina_infans* | 0.272 |
| *Anguilla_dieffenbachii* | *Avocettina_infans* | 0.234 |
| *Anguilla_interioris* | *Avocettina_infans* | 0.235 |
| *Anguilla_japonica* | *Avocettina_infans* | 0.254 |
| *Anguilla_marmorata* | *Avocettina_infans* | 0.279 |
| *Anguilla_megastoma* | *Avocettina_infans* | 0.271 |
| *Anguilla_mossambica* | *Avocettina_infans* | 0.250 |

**Table S1.** (Continued)

| *Anguilla_obscura* | *Avocettina_infans* | 0.243 |
| --- | --- | --- |
| *Anguilla_reinhardtii* | *Avocettina_infans* | 0.263 |
| *Anguilla_rostrata* | *Avocettina_infans* | 0.199 |
| *Anguilla_luzonensis* | *Avocettina_infans* | 0.282 |
| *Anguilla_australis_australis* | *Labichthys_carinatus* | 0.263 |
| *Anguilla_australis_schmidtii* | *Labichthys_carinatus* | 0.245 |
| *Anguilla_bicolor_bicolor* | *Labichthys_carinatus* | 0.272 |
| *Anguilla_bicolor_pacifica* | *Labichthys_carinatus* | 0.272 |
| *Anguilla_bengalensis_labiata* | *Labichthys_carinatus* | 0.280 |
| *Anguilla_bengalensis_bengalensis* | *Labichthys_carinatus* | 0.271 |
| *Anguilla_anguilla* | *Labichthys_carinatus* | 0.246 |
| *Anguilla_borneensis* | *Labichthys_carinatus* | 0.282 |
| *Anguilla_celebesensis* | *Labichthys_carinatus* | 0.274 |
| *Anguilla_dieffenbachii* | *Labichthys_carinatus* | 0.235 |
| *Anguilla_interioris* | *Labichthys_carinatus* | 0.236 |
| *Anguilla_japonica* | *Labichthys_carinatus* | 0.265 |
| *Anguilla_marmorata* | *Labichthys_carinatus* | 0.280 |
| *Anguilla_megastoma* | *Labichthys_carinatus* | 0.272 |
| *Anguilla_mossambica* | *Labichthys_carinatus* | 0.260 |
| *Anguilla_obscura* | *Labichthys_carinatus* | 0.244 |
| *Anguilla_reinhardtii* | *Labichthys_carinatus* | 0.264 |
| *Anguilla_rostrata* | *Labichthys_carinatus* | 0.208 |
| *Anguilla_luzonensis* | *Labichthys_carinatus* | 0.283 |
| *Anguilla_australis_australis* | *Heteroconger_hassi* | 0.264 |
| *Anguilla_australis_schmidtii* | *Heteroconger_hassi* | 0.282 |
| *Anguilla_bicolor_bicolor* | *Heteroconger_hassi* | 0.311 |
| *Anguilla_bicolor_pacifica* | *Heteroconger_hassi* | 0.321 |
| *Anguilla_bengalensis_labiata* | *Heteroconger_hassi* | 0.300 |
| *Anguilla_bengalensis_bengalensis* | *Heteroconger_hassi* | 0.291 |
| *Anguilla_anguilla* | *Heteroconger_hassi* | 0.282 |
| *Anguilla_borneensis* | *Heteroconger_hassi* | 0.303 |
| *Anguilla_celebesensis* | *Heteroconger_hassi* | 0.240 |
| *Anguilla_dieffenbachii* | *Heteroconger_hassi* | 0.275 |

**Table S1.** (Continued)

| *Anguilla_interioris* | *Heteroconger_hassi* | 0.274 |
| --- | --- | --- |
| *Anguilla_japonica* | *Heteroconger_hassi* | 0.294 |
| *Anguilla_marmorata* | *Heteroconger_hassi* | 0.310 |
| *Anguilla_megastoma* | *Heteroconger_hassi* | 0.255 |
| *Anguilla_mossambica* | *Heteroconger_hassi* | 0.275 |
| *Anguilla_obscura* | *Heteroconger_hassi* | 0.301 |
| *Anguilla_reinhardtii* | *Heteroconger_hassi* | 0.283 |
| *Anguilla_rostrata* | *Heteroconger_hassi* | 0.283 |
| *Anguilla_luzonensis* | *Heteroconger_hassi* | 0.293 |
| *Anguilla_australis_australis* | *Ariosoma_shiroanago* | 0.383 |
| *Anguilla_australis_schmidtii* | *Ariosoma_shiroanago* | 0.373 |
| *Anguilla_bicolor_bicolor* | *Ariosoma_shiroanago* | 0.344 |
| *Anguilla_bicolor_pacifica* | *Ariosoma_shiroanago* | 0.334 |
| *Anguilla_bengalensis_labiata* | *Ariosoma_shiroanago* | 0.325 |
| *Anguilla_bengalensis_bengalensis* | *Ariosoma_shiroanago* | 0.335 |
| *Anguilla_anguilla* | *Ariosoma_shiroanago* | 0.383 |
| *Anguilla_borneensis* | *Ariosoma_shiroanago* | 0.316 |
| *Anguilla_celebesensis* | *Ariosoma_shiroanago* | 0.335 |
| *Anguilla_dieffenbachii* | *Ariosoma_shiroanago* | 0.344 |
| *Anguilla_interioris* | *Ariosoma_shiroanago* | 0.325 |
| *Anguilla_japonica* | *Ariosoma_shiroanago* | 0.354 |
| *Anguilla_marmorata* | *Ariosoma_shiroanago* | 0.325 |
| *Anguilla_megastoma* | *Ariosoma_shiroanago* | 0.326 |
| *Anguilla_mossambica* | *Ariosoma_shiroanago* | 0.355 |
| *Anguilla_obscura* | *Ariosoma_shiroanago* | 0.334 |
| *Anguilla_reinhardtii* | *Ariosoma_shiroanago* | 0.355 |
| *Anguilla_rostrata* | *Ariosoma_shiroanago* | 0.364 |
| *Anguilla_luzonensis* | *Ariosoma_shiroanago* | 0.375 |
| *Anguilla_australis_australis* | *Conger_myriaster* | 0.348 |
| *Anguilla_australis_schmidtii* | *Conger_myriaster* | 0.329 |
| *Anguilla_bicolor_bicolor* | *Conger_myriaster* | 0.328 |
| *Anguilla_bicolor_pacifica* | *Conger_myriaster* | 0.318 |
| *Anguilla_bengalensis_labiata* | *Conger_myriaster* | 0.371 |

**Table S1.** (Continued)

| *Anguilla_bengalensis_bengalensis* | *Conger_myriaster* | 0.360 |
| --- | --- | --- |
| *Anguilla_anguilla* | *Conger_myriaster* | 0.351 |
| *Anguilla_borneensis* | *Conger_myriaster* | 0.351 |
| *Anguilla_celebesensis* | *Conger_myriaster* | 0.341 |
| *Anguilla_dieffenbachii* | *Conger_myriaster* | 0.340 |
| *Anguilla_interioris* | *Conger_myriaster* | 0.360 |
| *Anguilla_japonica* | *Conger_myriaster* | 0.320 |
| *Anguilla_marmorata* | *Conger_myriaster* | 0.319 |
| *Anguilla_megastoma* | *Conger_myriaster* | 0.340 |
| *Anguilla_mossambica* | *Conger_myriaster* | 0.312 |
| *Anguilla_obscura* | *Conger_myriaster* | 0.328 |
| *Anguilla_reinhardtii* | *Conger_myriaster* | 0.360 |
| *Anguilla_rostrata* | *Conger_myriaster* | 0.339 |
| *Anguilla_luzonensis* | *Conger_myriaster* | 0.349 |
| *Anguilla_australis_australis* | *Nettastoma_parviceps* | 0.294 |
| *Anguilla_australis_schmidtii* | *Nettastoma_parviceps* | 0.276 |
| *Anguilla_bicolor_bicolor* | *Nettastoma_parviceps* | 0.311 |
| *Anguilla_bicolor_pacifica* | *Nettastoma_parviceps* | 0.301 |
| *Anguilla_bengalensis_labiata* | *Nettastoma_parviceps* | 0.310 |
| *Anguilla_bengalensis_bengalensis* | *Nettastoma_parviceps* | 0.310 |
| *Anguilla_anguilla* | *Nettastoma_parviceps* | 0.273 |
| *Anguilla_borneensis* | *Nettastoma_parviceps* | 0.275 |
| *Anguilla_celebesensis* | *Nettastoma_parviceps* | 0.276 |
| *Anguilla_dieffenbachii* | *Nettastoma_parviceps* | 0.301 |
| *Anguilla_interioris* | *Nettastoma_parviceps* | 0.293 |
| *Anguilla_japonica* | *Nettastoma_parviceps* | 0.284 |
| *Anguilla_marmorata* | *Nettastoma_parviceps* | 0.330 |
| *Anguilla_megastoma* | *Nettastoma_parviceps* | 0.321 |
| *Anguilla_mossambica* | *Nettastoma_parviceps* | 0.301 |
| *Anguilla_obscura* | *Nettastoma_parviceps* | 0.301 |
| *Anguilla_reinhardtii* | *Nettastoma_parviceps* | 0.293 |
| *Anguilla_rostrata* | *Nettastoma_parviceps* | 0.272 |

**Table S1.** (Continued)

| *Anguilla_luzonensis* | *Nettastoma_parviceps* | 0.322 |
| --- | --- | --- |
| Summary | Minimum | 0.132 (13.2%) |
|  | Maximum | 0.332 (33.2%) |
|  | Average | 0.227 (22.7%) |
